# Apical cardiac hydatid cyst compressing the left ventricle: An uncommon manifestation of echinococcosis

**DOI:** 10.1016/j.idcr.2026.e02517

**Published:** 2026-02-12

**Authors:** Salma El Manir, Amine Allam, Noureddine Atmani, Amine Meskine, Younes Moutakiallah

**Affiliations:** a Department of Cardiac Surgery, Mohammed V Military Teaching Hospital, Rabat, Morocco; b Department of Anesthesiology and Intensive Care, Mohammed V Military Teaching Hospital, Rabat, Morocco

**Keywords:** Hydatid cyst, *Echinococcus granulosus*, Cardiac echinococcosis, Pericardial cyst, Cardiopulmonary bypass

## Abstract

**Background:**

Cardiac hydatidosis is a rare but serious manifestation of *Echinococcus granulosus* infection, accounting for less than 2 % of all hydatid disease cases. Ventricular involvement is exceptional, and cyst degeneration can mimic infection. Prompt recognition and surgical intervention are essential to prevent complications such as rupture, tamponade, or coronary compression.

**Case presentation:**

A 36-year-old woman with prior hepatic hydatid cystectomy presented with progressive dyspnea. Echocardiography and CT revealed a large cystic lesion compressing the left ventricular apex. The cyst was completely excised under cardiopulmonary bypass without aortic cross-clamping. Histopathology confirmed *E. granulosus*, and the postoperative course was uneventful.

**Discussion:**

Cardiac hydatid cysts can remain silent until they reach a significant size or undergo secondary changes. Diagnosis relies on echocardiography and CT imaging. Radical surgical excision under cardiopulmonary bypass, combined with albendazole therapy, ensures complete recovery and prevents recurrence.

**Conclusion:**

Hydatid disease should be considered in the differential diagnosis of any cystic cardiac lesion in endemic areas. Early surgical management remains crucial to avoid potentially fatal complications.

## Introduction

Hydatid disease is a zoonotic infection caused by the larval stage of *Echinococcus granulosus* [1]. Despite significant progress in prevention and veterinary control, it remains a major public health issue in endemic regions, particularly in developing countries. The liver and lungs are the most commonly affected organs, representing nearly 90 % of all cases, while cardiac involvement is reported in less than 2 % of patients with hydatidosis [2].

Within the heart, the left ventricle is most frequently affected because of its rich coronary blood supply, followed by the right ventricle, interventricular septum, and atria [3]. Pericardial localization is exceptional and may result from direct extension or hematogenous spread. Infection or rupture of a cardiac hydatid cyst can lead to serious complications such as anaphylaxis, tamponade, embolization, or sudden death [4].

We report an unusual case of a degenerating apical hydatid cyst compressing the left ventricle in a young woman with a prior history of hepatic hydatid disease. The case emphasizes the importance of considering hydatid etiology in any cystic cardiac lesion, particularly in endemic areas, and highlights the role of timely surgical management in ensuring accurate diagnosis and optimal outcomes.

## Case report

A 36-year-old woman presented with progressive exertional dyspnea over the past several weeks. She had undergone surgical resection of a hepatic hydatid cyst one year earlier. Physical examination revealed normal heart sounds without murmurs and no peripheral edema. Laboratory tests were unremarkable, and eosinophil count was within normal range.

**Fig. 1:**
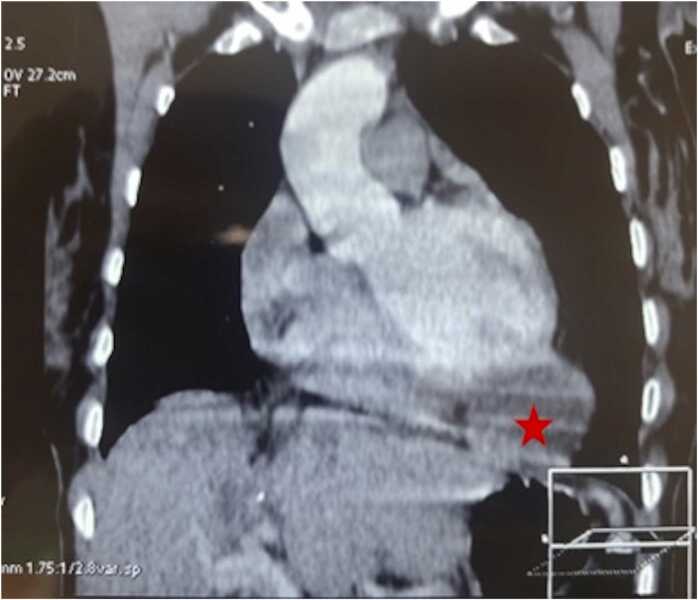
Thoracic computed tomography (coronal reconstruction) showing a well-defined cystic lesion (red star) located at the apical region of the left ventricle.

Transthoracic echocardiography demonstrated a large, well-defined cystic mass adherent to the anterolateral wall of the left ventricle, measuring approximately 74 × 51 mm. The lesion was heterogeneous with internal echolucent areas, suggestive of a hydatid cyst. Thoracic computed tomography confirmed a 67 mm cystic lesion adjacent to the apex of the left ventricle, exerting an external compressive effect (Fig. 1). Abdominal CT showed postoperative changes of partial hepatectomy without evidence of recurrence.

**Fig. 2:**
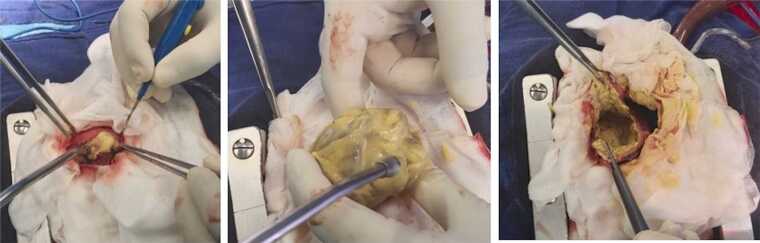
Intraoperative views: (A) Protective pads soaked with povidone–iodine placed around the cyst before controlled aspiration. (B) Macroscopic aspect of the cystic content during evacuation. (C) Open cyst cavity after evacuation and irrigation with scolicidal solutions.

Surgical exploration through median sternotomy was performed under cardiopulmonary bypass without aortic cross-clamping. Intraoperatively, a large cystic mass was found arising from the apical region of the left ventricle and compressing the ventricular wall. The lesion was in close contact with the distal portion of the left anterior descending coronary artery, without evidence of ischemia. Povidone–iodine–soaked pads were placed around the cyst to prevent dissemination of parasitic material (Fig. 2).

**Fig. 3:**
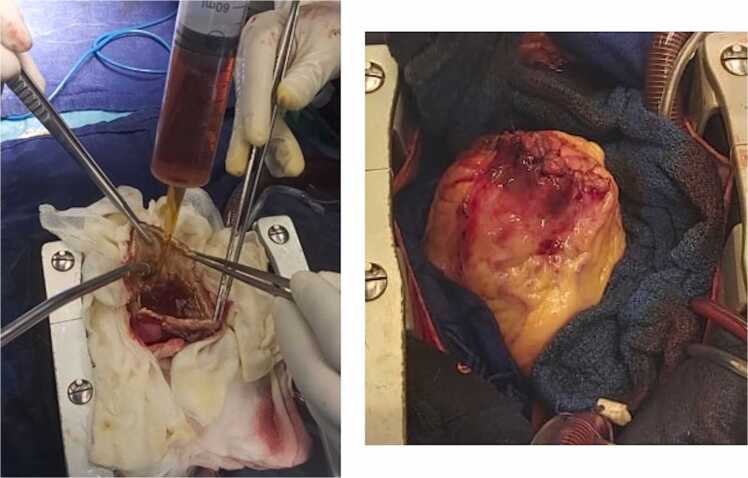
Intraoperative views: (A) Irrigation of the cystic cavity with scolicidal solutions before closure. (B) Final aspect after complete excision and closure of the residual cavity.

Upon incision, turbid fluid was aspirated, and the cystic content was completely evacuated. The cavity was irrigated with 20 % hypertonic saline and povidone–iodine solution, then closed by two layers of running sutures to prevent aneurysmal dilatation of the ventricular wall (Fig. 3).

**Fig. 4:**
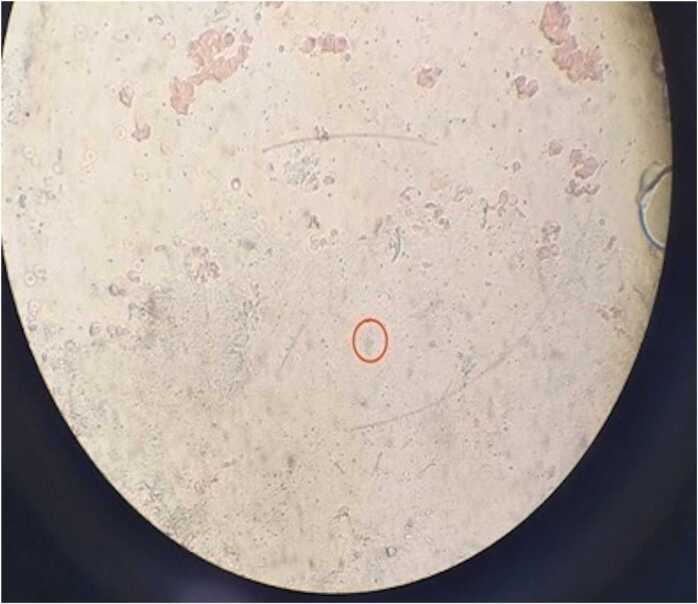
Histopathological examination showing laminated acellular membranes characteristic of *Echinococcus granulosus* (microscopic view, original magnification ×40).

Bacteriological analysis of the cystic fluid showed no bacterial growth. Histopathological examination confirmed the presence of vesicles typical of *Echinococcus granulosus* (Fig. 4).

The postoperative course was uneventful. The patient was extubated on the first postoperative day and discharged home on the eighth day without complications. She was treated with oral albendazole (10 mg/kg/day) for one month to prevent recurrence and remained asymptomatic at one-month follow-up.

## Discussion

Hydatid disease is a parasitic infection caused by the larval stage of *Echinococcus granulosus,* most frequently affecting the liver and lungs. Cardiac involvement is exceptionally rare, accounting for only 0.5 %–2 % of all hydatid disease cases, but it carries a high risk of severe and potentially fatal complications [1]. Within the heart, the left ventricle is the most commonly involved site due to its rich coronary blood supply, followed by the right ventricle, interventricular septum, atria, and, less commonly, the pericardium [2].

The clinical presentation of cardiac hydatid cysts is highly variable and depends on cyst size, location, and the presence of complications. Reported manifestations include chest pain, progressive dyspnea, palpitations, conduction disturbances, or embolic phenomena [3]. However, many patients remain asymptomatic for long periods, until the cyst degenerates, or ruptures, leading to pericardial effusion, cardiac tamponade, anaphylactic shock, and sudden death [4], [5]. Recent case reports have highlighted that even isolated intramyocardial cysts, particularly those involving the interventricular septum, may lead to significant hemodynamic compromise depending on their anatomical relationships [6].

In the present case, the cyst was located at the apical region of the left ventricle, producing localized compression of the ventricular wall and close contact with the distal left anterior descending coronary artery, without evidence of myocardial ischemia. The cyst contained turbid fluid but bacteriological culture was sterile. Such sterile degeneration with turbid content, sometimes mistaken for infection, has been previously described in the literature and may lead to diagnostic confusion [7].

Echocardiography remains the first-line imaging modality for the diagnosis of cardiac hydatid disease, allowing assessment of cyst morphology, mobility, and hemodynamic impact [7]. Computed tomography and magnetic resonance imaging provide complementary information by precisely defining anatomical relationships with myocardial tissue, coronary arteries, and pericardial structures, and are particularly useful for surgical planning [6]. Serological tests can support the diagnosis but are not always positive, particularly in isolated cardiac involvement.

Surgical excision remains the treatment of choice for cardiac hydatid cysts and should be performed promptly to prevent rupture and dissemination. Cardiopulmonary bypass facilitates controlled resection and minimizes the risk of intraoperative spillage. A critical step in surgical management is sterilization of the cystic cavity using scolicidal agents. Hypertonic saline, typically at a concentration of 20 %, is a well-established scolicidal solution that effectively inactivates protoscolices and reduces the risk of local or systemic dissemination. Protection of the operative field with hypertonic saline–soaked pads has been widely reported and is considered an essential precaution during cardiac hydatid surgery [8], [9].

Adjunctive antihelminthic therapy with albendazole is recommended in the perioperative period to minimize the risk of recurrence. Favorable outcomes have consistently been reported when complete surgical excision is combined with appropriate medical therapy and long-term follow-up [2], [9]. In our patient, complete surgical excision under cardiopulmonary bypass without cross-clamping, followed by albendazole therapy, resulted full clinical recovery.

This case underlines the importance of considering cardiac hydatid cysts in the differential diagnosis of cystic cardiac or pericardial lesions, especially in patients from endemic regions or with a history of hydatid disease. Early diagnosis and surgical management remain critical to preventing potentially fatal complications.

## Conclusion

Cardiac hydatid disease is a rare but serious manifestation of *Echinococcus granulosu*s infection. Although it represents less than 2 % of all hydatid cases, it carries a high risk of morbidity and mortality due to potential rupture, infection, or compression of vital cardiac structures. Diagnosis relies primarily on echocardiography and cross-sectional imaging, while serological tests may provide additional support.

Surgical excision remains the treatment of choice, ideally performed under cardiopulmonary bypass to minimize the risk of dissemination or rupture. Adjunctive antihelminthic therapy with albendazole further reduces the likelihood of recurrence. This case illustrates that early diagnosis and timely surgical management can lead to excellent outcomes with complete recovery.

## CRediT authorship contribution statement

**Amine Meskine:** Writing – review & editing, Conceptualization. **Younes Moutakiallah:** Writing – review & editing, Validation, Supervision. **Noureddine Atmani:** Writing – review & editing, Visualization. **Salma El Manir:** Writing – review & editing, Writing – original draft, Visualization, Resources, Project administration, Methodology, Investigation, Formal analysis, Data curation, Conceptualization. **Amine Allam:** Writing – review & editing.

## Ethical approval

Ethical approval was not required for this single-patient case report, in accordance with institutional policy, as written consent was obtained and no experimental intervention was performed.

## Author agreement

All authors have read and approved the final manuscript and agree with its submission to IDCases.

## Patient consent

Written informed consent was obtained from the patient for publication of this case report and the accompanying images. A copy of the written consent is available for review by the Editor-in-Chief of IDCases on request.

## Funding statement

This research received no specific grant from funding agencies in the public, commercial, or not-for-profit sectors.

## Declaration of Competing Interest

The authors declare no competing financial interests or personal relationships that could have influenced the work reported in this paper.

## Data Availability

No datasets were generated or analyzed for this report.
